# Microbiome Responses of Beef Steers to Rotational Grazing of Toxic Endophyte-Infected Tall Fescue Under Fall Conditions

**DOI:** 10.3390/ani16162497

**Published:** 2026-08-11

**Authors:** Ignacio M. Llada, Jeferson M. Lourenco, Mikayla M. Dycus, Utsav Lamichhane, Matthew K. Ross, Garret Suen, Dean P. Jones, Nicholas S. Hill, Nikolay M. Filipov

**Affiliations:** 1 Interdisciplinary Toxicology Program, University of Georgia, Athens, GA 30602, USA; ignacio.llada@uga.edu; 2 Department of Physiology and Pharmacology, University of Georgia, Athens, GA 30602, USA; 3 Department of Animal and Dairy Science, University of Georgia, Athens, GA 30602, USA; jefao@uga.edu (J.M.L.); madison.dycus@uga.edu (M.M.D.); utsav.lamichhane@uga.edu (U.L.); 4 Department of Comparative Biomedical Sciences, Mississippi State University, Starkville, MS 39762, USA; mross@cvm.msstate.edu; 5 Department of Bacteriology, University of Wisconsin, Madison, WI 53706, USA; gsuen@wisc.edu; 6 Department of Medicine, Division of Pulmonary, Allergy, Critical Care & Sleep Medicine, Emory University, Atlanta, GA 30322, USA; dpjones@emory.edu; 7 Department of Crop and Soil Sciences, College of Agriculture, University of Georgia, Athens, GA 30602, USA; nhill@uga.edu

**Keywords:** tall fescue, fescue toxicosis, *Epichloë coenophiala*, ergot alkaloids, microbiome

## Abstract

Fescue toxicosis results from ingestion of tall fescue infected with the ergot alkaloid (EA)-producing endophyte *Epichloë coenophiala*. The gastrointestinal microbiota is the first point of interaction with EAs and may be modulated by them. To investigate this, rumen and fecal samples were collected from steers grazing toxic (E+) or non-toxic fescue for 14 days, followed by a pasture switch. Microbial communities were characterized using 16S rRNA amplicon sequencing (bacterial V4, archaeal V6-V8 regions). Short-term E+ grazing did not affect alpha diversity in either matrix, but beta diversity differences were significant, though of small magnitude (rumen: R^2^ < 0.07; feces: R^2^ < 0.06), consistent with the overlap of communities between treatments. Relative abundance (RA) of dominant taxa remained unchanged, but low-abundance taxa were enriched in E+. In the rumen, these included proteolytic, amino acid decarboxylating, and methyl-compound forming microbes (e.g., *Clostridia*, *Prevotellaceae*, *Lachnospiraceae* (*Butyrivibrio*, *Eubacterium*), *Streptococcaceae*, and *Gamma*/*Alphaproteobacteria*), while fibrolytic taxa (e.g., *Ruminococcus flavefaciens*) declined. Hydrogenotrophic methanogens (*Methanobrevibacter_A*) decreased, while methylotrophic methanogens and hydrogen-utilizing bacteria (e.g., *Selenomonadaceae*) increased, indicating altered hydrogen flow in the rumen. Similar subtle but significant shifts were observed in fecal communities, including members of *Lachnospiraceae* (*Eubacterium*) and *Rikenellaceae* (*Mucinivorans*). Comparable RA of core (dominant) rumen and fecal microbiomes in steers previously exposed and never exposed to E+ fescue suggests no lasting effects on the dominant community. Short-term exposure to E+ tall fescue was associated with enrichment of low-abundance taxa whose previously described metabolic functions may contribute to maintaining rumen homeostasis under E+-induced dietary stress.

## Simple Summary

Tall fescue is one of the most widely used cool-season forages for livestock in the United States; however, its widespread infection by an ergot alkaloid (EA)–producing endophytic fungus makes it a serious threat to animal health. These mycotoxins cause peripheral and core vasoconstriction, disrupting thermoregulation and nutrient absorption. Interestingly, although the gastrointestinal microbiota represents the first point of interaction with EAs, little is known about how these toxins affect gut microbial communities. This study evaluated how short-term grazing on toxic fescue influences the rumen and fecal microbiota of steers. Animals grazed toxic (E+) or non-toxic fescue for 14 days, followed by a pasture switch, and microbial communities were characterized using 16S rRNA gene sequencing. The rumen and fecal core microbiome remained stable in relative abundance. However, some low-abundance microbes increased under E+ grazing conditions, including taxa previously reported to participate in protein metabolism and hydrogen utilization, while certain fiber-degrading microbes declined. The relative abundance of methane-producing archaea also shifted, a pattern consistent with altered ruminal hydrogen flow and/or substrate availability. These findings suggest that short-term exposure to E+ tall fescue is associated with enrichment of taxa whose known metabolic capabilities may help maintain rumen stability under E+ dietary stress and provide insight into how grazing E+ tall fescue reshapes gut microbiota and may influence the development of fescue toxicosis.

## 1. Introduction

Tall fescue (*Lolium arundinaceum*) is the predominant cool-season perennial grass in the United States, covering over 12–14 million hectares [1]. Fescue’s success as forage is largely attributed to its symbiosis with the endophyte fungus *Epichloë coenophiala*, which, through the production of secondary metabolites, enhances plant survival and fitness [2]. Unfortunately, some fungal metabolites, such as the ergot alkaloids (EA), are highly toxic for ruminants, leading to fescue toxicosis (FT), a disease characterized by reduced feed intake, poor weight gain, impaired thermoregulation, and decreased fertility [3]. While the clinical manifestations of FT are well characterized, far less is known about the unseen EA effects, particularly within the gastrointestinal tract microbiome.

Ruminants evolved as specialized herbivores in grassland ecosystems. This long-term evolutionary pressure shaped not only their unique digestive anatomy but also the development of a highly specialized gastrointestinal microbiome. This microbial community is adapted to break down complex plant materials and extract nutrients efficiently, supporting the ruminant’s overall energy requirements. The rumen microbiome provides the host with up to 70% of its energy and over 80% of its daily protein requirements [4], while the lower gut microbiome contributes up to ~10% of total metabolizable energy by fermenting nutrients that escape foregut digestion [5]; therefore, assessing the impact of grazing E+ tall fescue on both microbial ecosystems is necessary.

Beyond nutrient extraction, the microbiome has also adapted to metabolize a wide array of plant secondary compounds, effectively acting as a natural detoxification system that protects the host from potentially harmful dietary constituents [6]. Evidence indicates that EA, like ergovaline (EV), can be cleaved by ruminal microorganisms into less potent compounds (e.g., lysergic acid) [7,8] that are ultimately excreted from the body. In alignment, an in vitro study showed that incubating toxic fescue seed with ruminal fluid reduced its toxicity, as rats fed these incubated seeds gained more weight than those fed unfermented seed [9]. Another in vitro study demonstrated that certain rumen bacteria (e.g., *Prevotella* spp. and *Clostridium* spp.) are involved in EV degradation [10]. Altogether, these findings suggest that microbes play a key role in EA detoxification. At the same time, EA exposure may impose selective pressures on microbial populations, likely favoring those capable of detoxification and consequently altering community diversity. Supporting this, continuous toxic fescue grazing studies have been shown to alter the diversity of the gastrointestinal microbiome in steers [11,12] and cows [13]. Therefore, understanding whether these shifts represent adaptive responses that enhance detoxification or maladaptive changes that contribute to FT is essential for determining their role in the clinical manifestations of the disease.

Not all microbial processes benefit the host. Rumen methanogenic archaea convert fermentation by-products into methane, a gas that represents a loss of up to 12% of gross energy intake and contributes to greenhouse gas emissions [14]. Data on beef methane emissions and archaeal populations while grazing toxic fescue are limited. One study reported no difference in methane emissions between animals grazing toxic or endophyte-free pastures [15], whereas a more recent study found that *Methanobrevibacter*, the dominant methanogen, was reduced while methylotrophic methanogens (*Methanomethylophilaceae*) were increased in steers grazing fescue [16]. Although profiling the archaeal community does not determine whether methane emissions increase or decrease, identifying predominant archaeal genera can provide insight into the substrates available during toxic fescue exposure and link archaeal community dynamics with rumen fermentation patterns and the animal’s metabolic response to toxins (e.g., EA).

Extending the investigation of a trial where the focus was on EA kinetics and metabolome alterations [17,18], this study used next-generation 16S rRNA sequencing on rumen fluid and solids, as well as fecal samples of steers rotationally grazing toxic fescue to (a) characterize the bacterial and archaeal diversity and composition, (b) identify any residual effects on the microbiome following removal from toxic pastures, and (c) correlate ruminal and fecal microbial composition with EA and trace amine levels determined using targeted [17] and untargeted [18] analysis. This multi-compartment approach provides a comprehensive view of how short-term grazing of toxic fescue influences microbiota composition along the gastrointestinal tract.

## 2. Materials and Methods

### 2.1. Animals, Treatments, and Experimental Design

The study was conducted in the fall of 2023 (18th October to 15th November), on pastures located at the University of Georgia’s J. Phil Campbell Natural Resources Conservation Center (Watkinsville, GA, USA). Because tall fescue pastures in Georgia are extensively infected with the toxic endophyte, prior lifetime exposure of the animals to EA cannot be ruled out; however, for at least two weeks before the start of the trial, all animals were maintained exclusively on fescue-free hay. Consistent with this washout, a targeted EA analysis of the same animals, published elsewhere [17], detected only negligible EA concentrations in biological matrices before pasture placement. Following this preconditioning period, post-weaning steers (*n*=18; BW = 200.3 ± 4.1 kg) were blocked by weight and randomly assigned to 1.2 ha of tall fescue pastures containing a new novel, non-toxic endophyte (NE; Jesup MaxQ strain AR542, *n* = 6; 204.6 ± 7.2 kg), a toxic endophyte (E+; Jesup with wild-type endophyte, *n* = 6; 198.7 ± 8.1 kg), and endophyte-free fescue (E-; *n* = 6; 197.5 ± 7.0 kg), with a stocking density of 0.2 ha/steer. The steers were kept on these pastures for 14 days and switched as follows: those on E+ were switched to non-toxic pastures (E- or NE); six steers from non-toxic pastures (E- or NE) were switched to E+, and the remaining six steers stayed on non-toxic pastures (E- or NE). They remained on their newly assigned pastures for an additional 14 days. Rumen content and feces were collected before (pre), 2, 7, 14, 16, 21, and 28 days after pasture allocation. Fescue plants were collected on the first and the last day of the trial for endophyte and EA analyses. All samples were collected between 8:00 and 12:00.

### 2.2. Sample Collection and Processing

Hand-collected fresh fecal samples were placed in 50 mL conical tubes and stored on ice. Samples of ruminal content were collected as in [12] using a ruminal probe, which was washed between animals. To minimize salivary contamination, the first portion of aspirated rumen fluid was discarded, and only the subsequent fraction was retained for analysis. Approximately 50 mL of ruminal digesta was processed by passing it through four layers of sterile autoclaved cheesecloth to separate the liquid and solid fractions. Each fraction was transferred into sterile cryovials, immediately placed on dry ice for transport, and stored at −80 °C upon arrival at the laboratory until subsequent analyses.

### 2.3. Microbiome Sample Processing and Data Analysis

#### 2.3.1. DNA Extraction

Genomic DNA was isolated from feces and from rumen contents (solid and fluid fractions) following an adaptation of the protocol of Stevenson and Weimer [19], based on bead-beating disruption followed by phenol extraction (25:24:1 phenol:chloroform:isoamyl alcohol) [20]. Extracted DNA was resuspended in TE (Tris-EDTA) buffer, and concentrations were determined on a Qubit^®^ Fluorometer (Invitrogen, San Diego, CA, USA). To ensure the reliability of the procedure, a negative control (TE extraction buffer) was included alongside each extraction and subjected to the same amplification and sequencing protocols outlined below.

#### 2.3.2. DNA Amplification and Sequencing

Bacterial 16S rRNA gene amplification and sequencing were performed as described in [11,21]. Briefly, samples were diluted to 1 ng/μL for amplification, employing universal bacterial primers targeting the 16S rRNA gene variable region V4 [22]. Reactions of 25 μL contained 12.5 μL KAPA HiFi master mix (Kapa Biosystems, Wilmington, MA, USA), 5 μL template, 0.5 μL of each 10 μM primer (forward 5′-GTGCCAGCMGCCGCGGTAA-3′; reverse 5′-GGACTACHVGGGTWTCTAAT-3′), and 6.5 μL water. Negative PCR controls containing extraction buffer and primers were run on each plate. The thermal profile consisted of 95 °C for 3 min; 25 cycles of 95 °C for 30 s, 55 °C for 30 s, and 72 °C for 30 s; and 72 °C for 5 min. Amplicons were purified on 1% (wt/vol) low-melt agarose gels with a Zymoclean 96-well DNA recovery kit (Zymo Research, Irvine, CA, USA). Plates yielding visible bands in the negative controls were repeated until controls were clean. Purified amplicons were quantified on the Qubit and pooled equimolarly, and the pooled library was quality filtered and normalized prior to sequencing. Libraries were sequenced with custom primers [22] on an Illumina MiSeq using a 2 × 250 bp paired-end v2 kit (Illumina, San Diego, CA, USA). Archaeal 16S rRNA gene libraries targeted the V6-V8 region using a two-step primer approach described by Kittelmann et al. [23], with archaeal-specific primers Ar915aF (AGGAATTGGCGGGGGAGCAC) and Ar1386R (GCGGTGTGTGCAAGGAGC); all the subsequent procedures were the same as those described for bacteria.

#### 2.3.3. 16S rRNA Gene Sequence Processing and Bioinformatics Analysis

Bacterial and archaeal FASTQ files were processed in QIIME 2 v.2024.10 [24]. Quality control, paired-read merging, and chimera removal were performed with the DADA2 plugin [25], and reads were resolved into Amplicon Sequence Variants (ASVs) at 100% identity. Taxonomy was assigned with the feature-classifier plugin using a Naïve Bayes classifier trained on the Greengenes2 reference database [26]. Taxonomic data were further cleaned and summarized using the mbX package in R (version 4.4.2) [27]. Relative abundance of individual microbial taxa was summarized at different taxonomic levels ranging from phyla to species. In addition, samples were rarefied to a common sequencing depth of 9445 sequences/sample for calculation of alpha- and beta-diversity (Bray–Curtis distances). Alpha-diversity metrics included Shannon diversity index, Faith’s Phylogenetic Diversity, Pielou’s evenness, and the number of observed features (ASV).

### 2.4. Statistical Analysis

Individual taxonomic abundances and alpha-diversity indices were analyzed for statistical significance in R (version 4.4.2; R Core Team, Vienna, Austria). Mixed-effects models were fitted for all response variables using the lmerTest package, with animal ID included as a random effect to account for repeated measures. Fixed effects included day, treatment, and their interaction (day × treatment). All analyses were performed using Type III sums of squares. The full model was retained for all analyses regardless of the statistical significance of the interaction term, as the interaction was specified a priori based on the experimental design and to maintain a consistent analytical framework across all response variables. Data preprocessing was conducted using dplyr; least-squares means and pairwise comparisons were obtained using emmeans, and multiple-comparison adjustments were performed using multcomp. The same statistical procedures were used to examine differences in archaeal relative abundance at the genus level. Beta-diversity was assessed using Bray–Curtis dissimilarities. Group differences in microbial community composition were tested using permutational multivariate analysis of variance (PERMANOVA), implemented in QIIME 2 v.2024.10 via the qiime diversity beta-group-significance plugin, with statistical significance assessed using 999 permutations. To obtain effect size estimates, the corresponding distance matrices were exported and reanalyzed in R using the adonis2 function (vegan package) with 999 permutations. The coefficient of determination (R^2^), representing the proportion of total variation in microbial community composition explained by treatment, was obtained from the PERMANOVA output and reported alongside the *p*-value as a measure of effect size. The results were visualized in dimensional space using EMPeror. For all statistical tests, results were considered significant at *p* ≤ 0.05, with *p*-values between 0.05 and 0.10 treated as trends. Given the limited group size of this study (*n* = 6), PERMANOVA can detect marginal compositional differences as statistically significant and may be sensitive to heterogeneity of multivariate dispersion, which was not formally evaluated (e.g., using PERMDISP/betadisper) and thus remains a limitation. Therefore, beta-diversity results were interpreted with caution and in conjunction with the effect sizes and ordination patterns.

To assess pre- and post-pasture placement changes in the rumen fluid, solid, and fecal microbiome of E+ steers, only microbial taxa at the genus level with a relative abundance (RA) ≥ 0.3% at both sampling points (day 1, *n* = 6; day 14, *n* = 6) were included in the analysis. For each taxon, paired comparisons were performed using the Wilcoxon signed-rank test in R. Raw *p*-values were adjusted for multiple comparisons using the Benjamini–Hochberg false discovery rate (FDR), and statistical significance was determined based on the FDR-adjusted values. The same analytical workflow was used to assess archaeal RA pre- and post-pasture placement in E+ steers. To investigate potential microbial shifts in bacteria and archaea in steers newly exposed to toxic pasture during the second 14-day period, the same workflow was applied. In this case, the pre-exposure time point corresponded to day 14 (the last day on non-toxic pasture), and the post-exposure time point corresponded to day 28 (the last day on toxic pasture). Due to the somewhat limited sensitivity of the paired Wilcoxon signed-rank test (*n* = 6 pairs), statistical power across the large number of taxa tested was reduced. Accordingly, results with *p*-values between 0.05 and 0.10 are reported as trends and regarded as hypothesis-generating signals that require confirmation in larger cohorts rather than as definitive effects. The cutoff used in this analysis was applied to focus the paired comparison on the abundant, consistently detected taxa (the core microbiome), which are more reliable for paired comparisons than low-abundance taxa [28]. Applying a minimum RA threshold to distinguish core from rare taxa is common in microbiome studies and reduces the technical noise introduced by low-abundance taxa, although no universal cutoff exists and the specific value is in part conventional [29]. Low-abundance taxa (<0.3%) were not excluded from the study; they were analyzed separately using the mixed-effects model described above. During the first and second 14-day periods, repeated-measures correlations were performed using the rmcorr package in R [30]. This was done to assess relationships between ruminal and fecal microbes at the genus level and (a) EA concentration (ergovaline and lysergic acid), detected/quantified using a targeted approach [17], or (b) trace amines (tyramine and methyltyramine), detected using an untargeted approach [18], in the rumen fluid, saliva, urine, and plasma of E+ steers. Animal ID was used to account for repeated measurements within each animal. *p*-values were adjusted for multiple comparisons using the Benjamini–Hochberg false discovery rate (FDR), and correlations with FDR-adjusted *p* < 0.05 were considered significant.

## 3. Results

### 3.1. Plant Endophyte and Total Ergot Alkaloids (EA)

Endophyte presence averaged 70% in E+, 82% in NE, and 0% in E-. Total EA in whole-plant tissue was >6000 ppb in E+, and 0 ppb in NE and E-.

As a separate arm of this trial, the EA concentration (ergovaline and lysergic acid) was also assessed in multiple biological matrices, including rumen fluid, saliva, urine, and plasma. This approach provided the opportunity to investigate the EA dynamics across different matrices and confirmed that animals grazing E+ were consistently exposed to these compounds throughout the study period. For further details, refer to [17].

### 3.2. 16S rRNA Gene Sequencing

We generated 17,561,019 raw sequences, which resulted in 12,947,856 high-quality sequences after filtering. The average number of paired sequences per sample was 33,588 (range: 8580–83,312).

### 3.3. Alpha Diversity

No significant differences (*p* ≥ 0.3) in richness and diversity metrics were observed between E+ and the other groups in any of the analyzed matrices, either during the first 14 days or after the pasture switch (Supplementary Figure S1).

### 3.4. Beta Diversity

In rumen fluid, overall beta diversity showed a trend (PERMANOVA: R^2^ = 0.039, *p* = 0.09) during the first 14 days on E+ compared to the other groups, whereas no differences (*p* > 0.1) were observed between steers grazing E- or NE pastures. No differences (*p* > 0.5) were detected prior to pasture placement. Only on day 7, a treatment-by-day interaction was observed between E+ and E-, although it did not reach statistical significance (*p* = 0.06). On the remaining days, no significant interactions were detected (*p* > 0.2) between groups. During the second 14-day period (PERMANOVA: R^2^ = 0.052), a trend was also observed in overall beta diversity (*p* = 0.06) between steers newly exposed to E+ and the other groups. Additionally, there was a trend (*p* = 0.06) between steers previously exposed to E+ and those never exposed. By day of sampling, no differences were detected by groups (*p* > 0.6).

In the rumen solid, an overall significant difference (PERMANOVA: R^2^ = 0.052, *p* < 0.05) in beta diversity was observed during the first 14 days between E+ and the other groups. When analyzed by day of sampling, no differences (*p* > 0.1) were detected prior to pasture placement; however, on days 7 and 14, beta diversity was significantly different between the E+ and the other groups (*p* = 0.02). During the second 14 days, the overall PERMANOVA showed differences in beta diversity among groups (R^2^ = 0.071, *p* ≤ 0.001). Beta diversity of steers newly exposed to E+ fescue differed significantly from the other groups and also differed between steers previously exposed to E+ and those never exposed. By day of sampling, these differences were evident on day 16 (two days post-pasture placement), with no differences (*p* > 0.2) on the remaining days.

In feces, an overall significant difference (PERMANOVA: R^2^ = 0.044, *p* ≤ 0.03) in beta diversity was observed during the first 14 days between the E+ group and the rest. By day of sampling, no differences were detected prior to pasture placement (*p* > 0.1) and on day 2 (*p* > 0.4), while on days 7 and 14, there was a tendency (*p* = 0.08) to be different between the E+ and the other groups. During the second 14 days (PERMANOVA: R^2^ = 0.060), beta diversity of newly exposed steers to E+ pastures was significantly different compared to the other groups (*p* ≤ 0.001), while no difference (*p* > 0.1) was observed between steers previously exposed to E+ and those never exposed. By day of sampling, no differences were observed between groups (*p* ≥ 0.8).

In the rumen solids and feces, although a significant difference in beta diversity was detected, the principal coordinates analysis (PCoA) plots did not show a clear separation between groups. Given the low R^2^ values obtained in the different matrices analyzed, treatment accounted for only a small proportion (4–7%) of the total microbial variation, indicating that these statistically significant differences represent subtle rather than large-scale shifts in community composition. For more details on the overall beta diversity across matrices, refer to Supplementary Figure S2.

### 3.5. Specific Microbial Taxa

Throughout this section, numeric suffixes and letter designations appended to taxon names (e.g., *_A*, *_168226*) are Greengenes2 database-specific identifiers.

**Figure 1:**
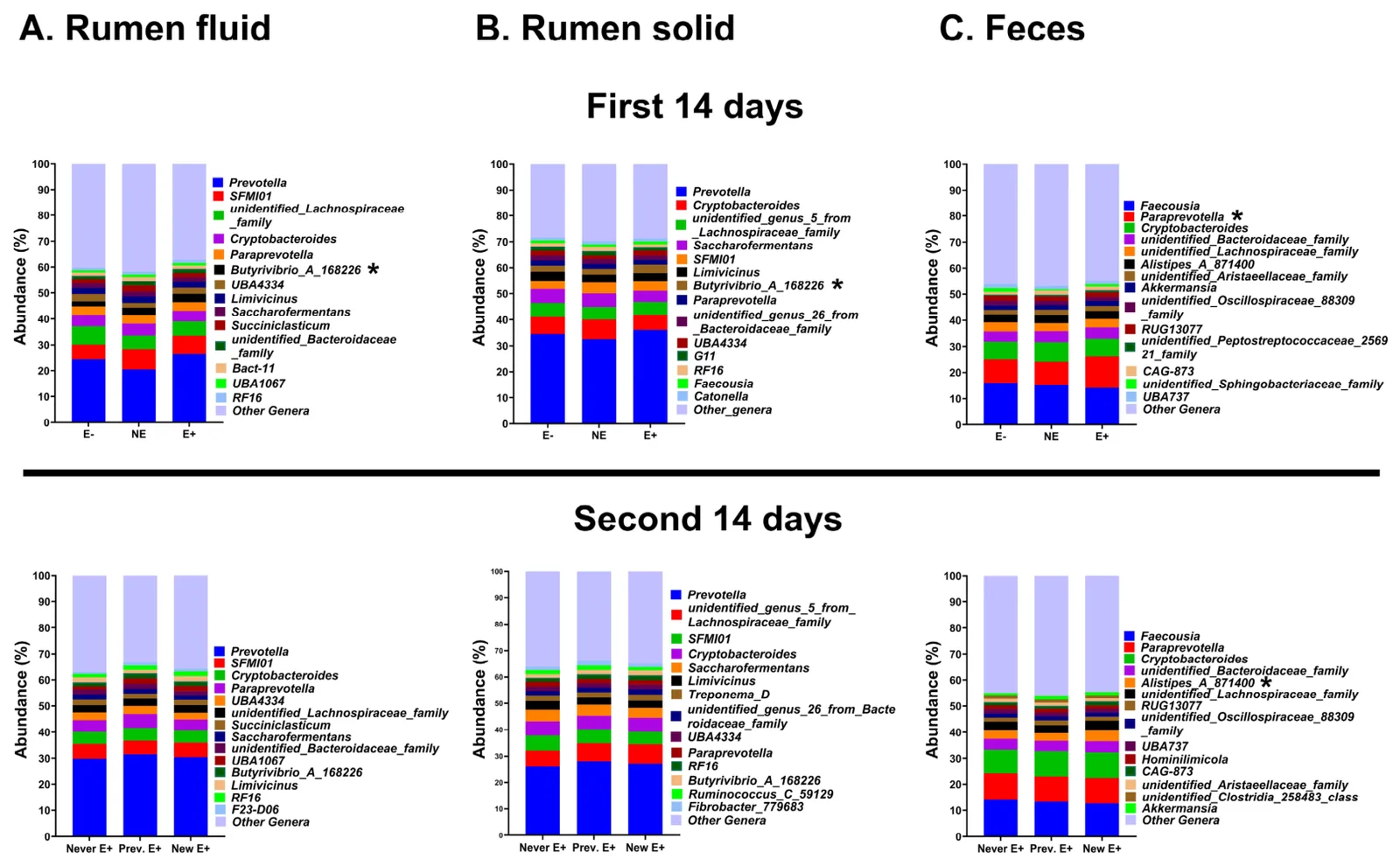
Relative abundance (%) of the top 15 genera in the (**A**) rumen fluid, (**B**) rumen solid, and (**C**) feces of Angus steers grazing on tall fescue infected with toxic endophyte (E+; *n* = 6), novel non-toxic endophyte (NE; *n* = 6), or endophyte-free (E-; *n* = 6) during the first 14 days (**upper panel**), and after switching treatments (**bottom panel**). (*) indicates genera that differed significantly in abundance between the E+ group and the other treatments (*p* ≤ 0.05).

Among microorganisms (MOs) with a relative abundance (RA) ≥ 0.3% in rumen fluid and solid, the phylum *Bacteroidota* predominated, followed by *Bacillota* (formerly *Firmicutes*), while at the family level, *Bacteroidaceae* and *Lachnospiraceae* were the most abundant (Supplementary Figure S3A,B). In feces, the microbial community was dominated at the phylum level by *Bacillota*, followed by *Bacteroidota*, while at the family level, *Oscillospiraceae* (formerly *Ruminococcaceae*) was the most abundant, followed by *Bacteroidaceae* (Supplementary Figure S3C). Among the top 15 taxa at phylum and family levels, no differences were detected between treatments (*p* ≥ 0.4) in any analyzed matrix (Supplementary Figure S3). At the genus level in the rumen fluid and solid, *Prevotella* was the most abundant across periods (Figure 1), but no differences were detected between treatments (*p* > 0.4). Among the top 15 genera, only *Butyrivibrio_A_168226* differed significantly (*p* = 0.02) among treatments. In rumen fluid during the first 14 days, its RA was higher in E+ steers (3.2%) compared with E- (2.0%; *p* = 0.02), but not significantly different from NE steers (2.7%) (Figure 1); in rumen solid, the same genus was also higher in E+ steers (3.0%) compared with E- (2.2%) and NE (2.1%; *p* = 0.04) (Figure 1). During the second 14-day period, none of the top 15 genera differed between groups (*p* ≥ 0.2) (Figure 1). At the genus level in feces, the community was dominated by *Faecousia*, followed by *Paraprevotella* (Figure 1). Among the top 15 genera, only the RA of *Paraprevotella* was significantly greater (*p* ≤ 0.05) in E+ steers (11%) compared with E- (8.7%) or NE (8%) during the first 14 days (Figure 1). In contrast, during the second 14 days, only the RA of *Alistipes_A_871400* differed among groups (Figure 1), being higher (*p* ≤ 0.05) in steers newly exposed to E+ (4.1%) compared with those previously exposed (3.0%) or never exposed (3.2%), while no difference was detected between the latter two groups (*p* > 0.9).

Among MOs with RA < 0.3%, in the rumen fluid, some were significantly affected in E+ steers. Most changes were observed within the phylum *Bacillota*, predominantly in the class *Clostridia*, including members of the families *Lachnospiraceae* (e.g., *Eubacterium_G*, *Butyrivibrio_A*) and *Ruminococcaceae* (*Ruminococcus_C*). Some members of these taxa decreased in RA (e.g., *UBA1248*, *PeH17 sp900542285*, *Ruminococcus_C flavefaciens_F*), while others increased (e.g., *Butyrivibrio_A*, *Eubacterium_G*). Other affected phyla included *Bacteroidota* (e.g., *UBA6382*) and *Pseudomonadota*, with members belonging to the classes *Gammaproteobacteria* (e.g., *Rhodocyclaceae*) and *Alphaproteobacteria,* which increased in RA. Minor, but significant changes were also observed in other phyla, including increases in *Bacillota_I* (*Streptococcus infantarius*, *UBA6985*). In the rumen solid, most of the low-abundance affected taxa belonged to the phylum *Bacillota*, primarily the class *Clostridia*, and included members of the families *Lachnospiraceae* (e.g., *XBB2008*, *Butyrivibrio_A_168226*, *Eubacterium_G_142752*) and *Ruminococcaceae* (*Ruminococcus_C_58660*). Some of these taxa decreased in RA (*G11*), while others increased (*XBB2008*, *Butyrivibrio_A_168226*, *Eubacterium_G_142752*). Minor *Bacillota_I* taxa (*RUG12783*) and *Bacillota_C* (*Selenomonas_B ruminantium_A*) also increased. A potential proteolytic *Pseudomonadota* taxon (*Rhodocyclaceae*) also increased in RA. In feces, most of these minor taxa belonged to the phylum *Bacillota*, primarily the class *Clostridia*, family *Lachnospiraceae* (*Eubacterium_G_142752*), as well as *Bacteroidota*, family *Bacteroidaceae* (*Paraprevotella*), which increased in RA. For additional information on rumen and fecal microbes with RA below 0.3% that were significantly altered in E+ steers, refer to Table 1.

**Figure 2:**
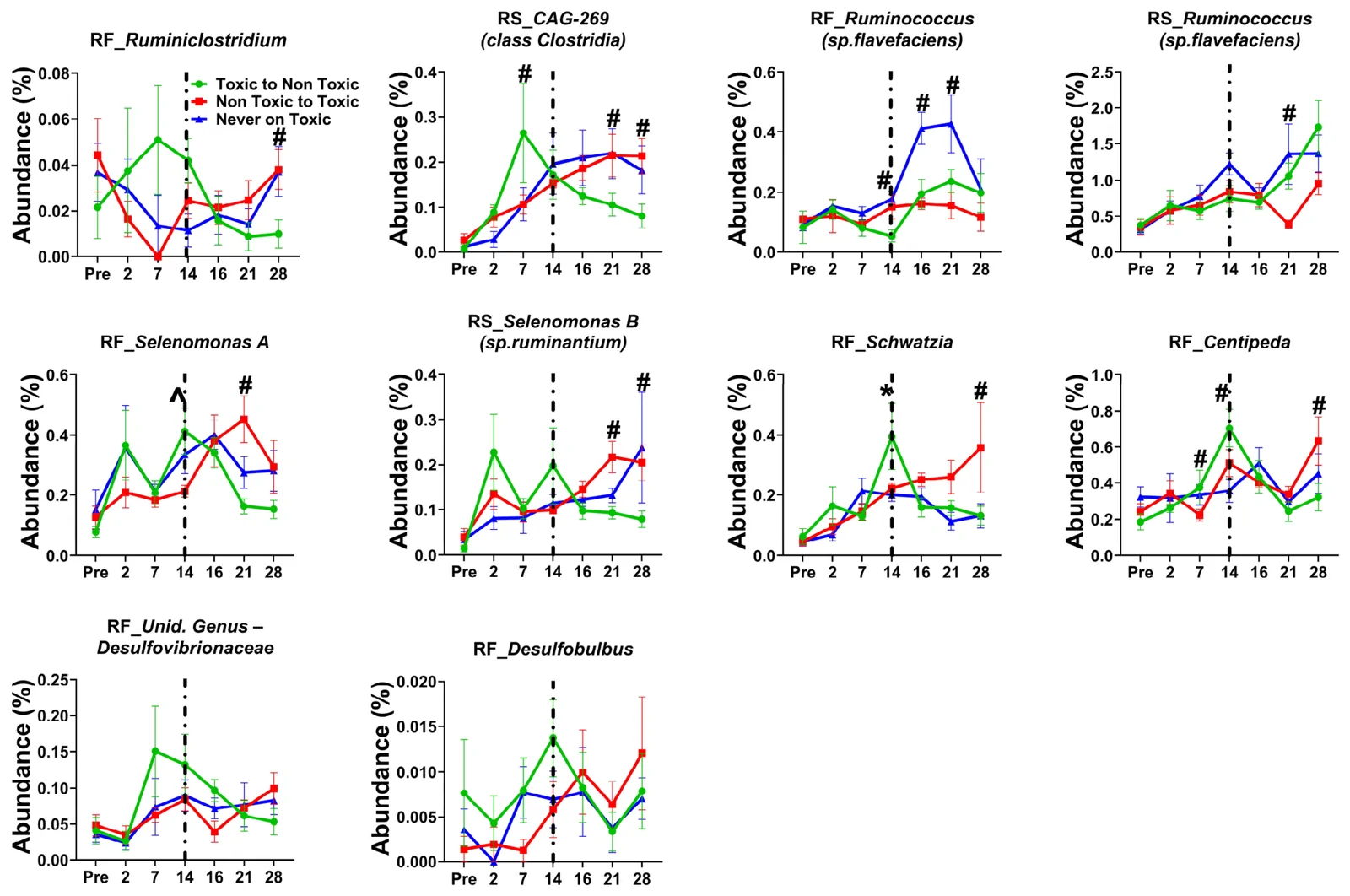
Relative abundance (%) across sampling days for selected bacterial taxa that exhibited a specific pattern in the rumen fluid (RF) or solid (RS). (#) indicates a significant difference (*p* ≤ 0.05) between the E+ group and one of the other groups, (*) indicates a significant difference between E+ and the rest (*p* ≤ 0.05), while (^) indicates trends (*p* ≥ 0.05, *p* < 0.1). Data are presented as mean ± SEM; *n* = 6 animals per group. Dashed line marks pasture treatment switch on day 14.

Some bacteria that exhibited a significant effect of treatment and/or day × treatment followed a specific pattern: their RA increased, either significantly or numerically, during grazing on E+ fescue and decreased following removal from toxic pastures. In rumen fluid, these taxa spanned multiple phyla, including *Bacillota*, *Bacteroidota*, *Desulfobacterota*, and *Pseudomonadota*. Within *Bacillota*, they included fiber degraders (*Ruminiclostridium_E siraeum*), carbohydrate fermenters (*UBA2730 sp900320505*, *UBA6985 sp900314465*), and succinate/propionate producers (*Selenomonas_A_42750*, *Schwartzia succinivorans*, *Centipeda*). *Bacteroidota* taxa (*UBA932*) were mainly polysaccharide degraders, while *Desulfobacterota* (*Desulfovibrionaceae*, *Desulfobulbus oralis*) were sulfate-reducing bacteria. In the rumen solid, these taxa included members of the phylum *Bacillota*, primarily the families *Lachnospiraceae* (*G11*), *TANB77* (*CAG-269*, formerly annotated as *Clostridium* sp. *CAG:269* in NCBI), and *Selenomonadaceae* (*Selenomonas_B ruminantium_A*); *Bacteroidota*, family *Bacteroidaceae* (*UBA4334*); and *Pseudomonadota*, family *Rhodocyclaceae* (genus unclassified). In feces, members of phylum *Bacillota*, family *Lachnospiraceae* (*Eubacterium_G_142752*) and *Anaerovoracaceae* (*Eubacterium_T_231757*), as well as *Bacillota_I* (*RF39 CAG-914*, *ML615J-28 UBA2253*) and *Bacteroidota*, families *Muribaculaceae* (*CAG-873*) and *Rikenellaceae* (*Mucinivorans*), followed the same pattern. Selected microbes that followed this specific pattern in the rumen fluid and solid are shown in Figure 2.

**Figure 3:**
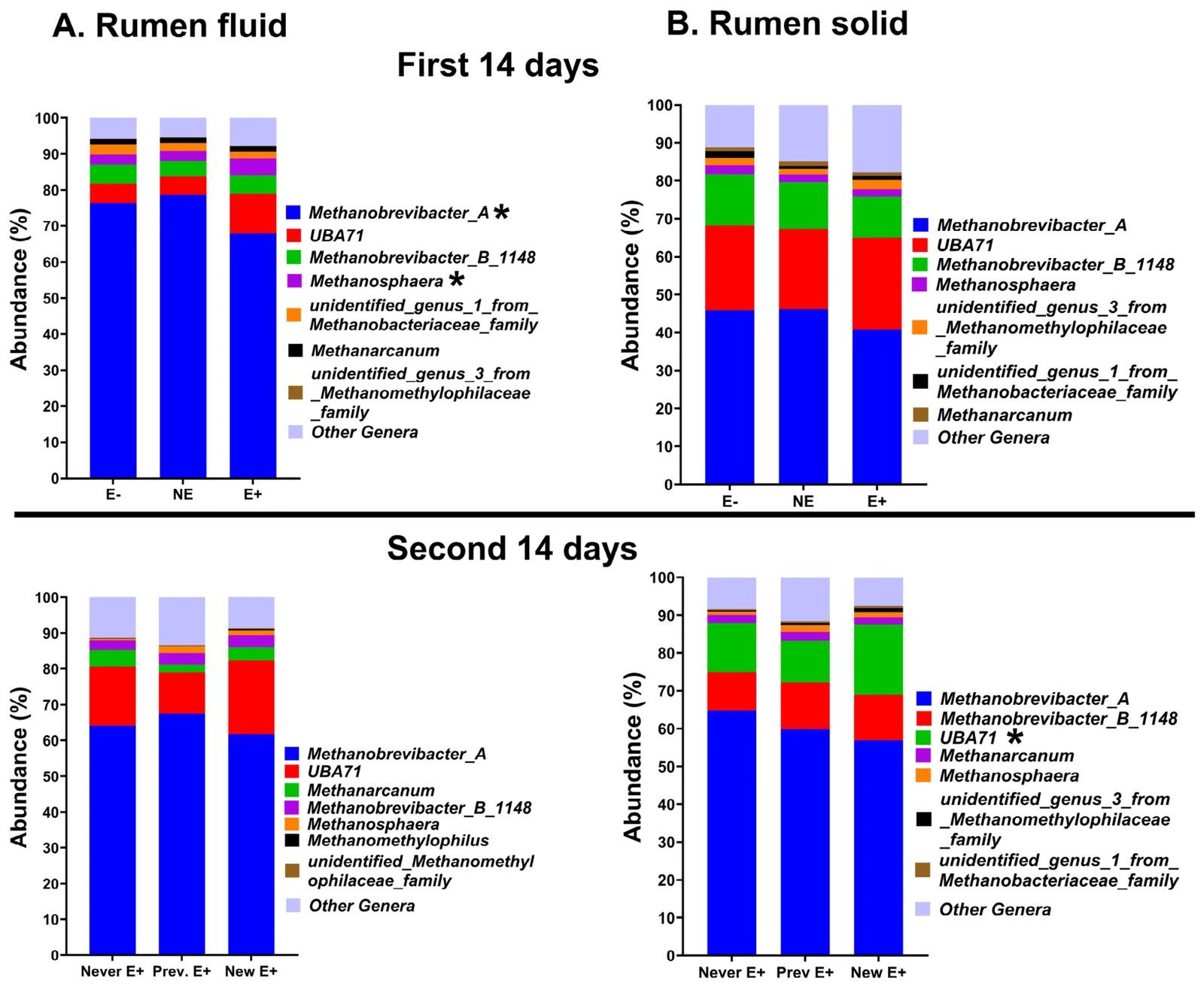
Relative abundance (%) of dominant archaeal genera in the (**A**) rumen fluid, and (**B**) rumen solid of Angus steers grazing on tall fescue infected with toxic endophyte (E+; *n* = 6), novel non-toxic endophyte (NE; *n* = 6), or endophyte-free (E-; *n* = 6) during the first 14 days (**upper panel**), and after switching treatments (**bottom panel**). (*) indicates genera that differed significantly in abundance between the E+ group and the other treatments (*p* ≤ 0.05).

**Figure 4:**
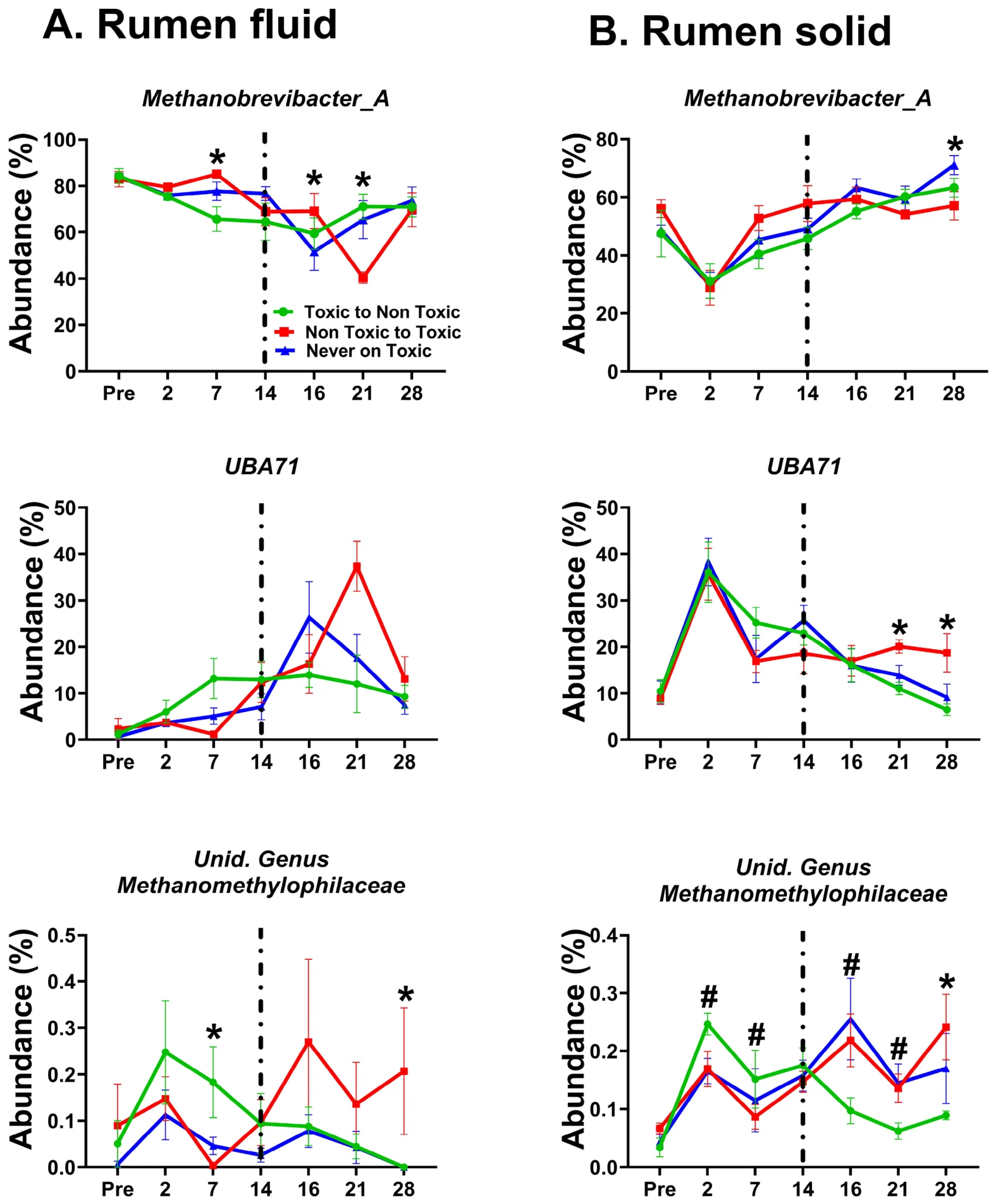
Relative abundance (%) across sampling days for selected Archaea in rumen fluid (**A**) and rumen solid (**B**). (#) indicates a significant difference (*p* ≤ 0.05) between the E+ group and one of the other groups, while (*) indicates a significant difference between E+ and the rest (*p* ≤ 0.05). Data are presented as mean ± SEM; *n* = 6 animals per group. Dashed line marks pasture treatment switch on day 14.

Regarding the archaeal population, the genus *Methanobrevibacter_A* dominated in both rumen fluid and solid (Figure 3). During the first 14 days, its RA was lower in E+ steers (68%) compared with E- (76%) and NE (80%) (*p* ≤ 0.05) in rumen fluid, while in rumen solid it was numerically lower in E+ steers (41%) compared with E- and NE (both 46%) (Figure 3). The second most abundant genus, *UBA71* (a methylotrophic methanogen within the order *Methanomassiliicoccales*, family *Methanomethylophilaceae* [31], that generates methane by reducing methyl compounds, such as methanol and methylamines, using H_2_ as the electron donor [32,33]), was numerically higher in E+ steers (rumen fluid: 11% vs. 5% in E- and NE; rumen solid: 24% vs. 22% and 21%, respectively) (Figure 3). In the rumen fluid, the only archaeal taxon showing an overall significant difference in RA was *Methanosphaera*, which was higher in E+ (5%) compared with E- (3%) and NE (3%) (*p* ≤ 0.02) (Figure 3). During the second 14 days, no differences were detected among treatments (*p* ≥ 0.2); though the trend in rumen fluid, with decreased *Methanobrevibacter_A* and increased *UBA71* in steers newly exposed to E+, persisted numerically (Figure 3). In the rumen solid, *Methanobrevibacter_A* remained numerically lower in steers newly exposed to E+ (57%) compared with those previously (60%) or never exposed (65%) to E+, while *UBA71* was significantly higher in newly exposed animals (19%) than in previously exposed (11%) or never exposed (13%) (*p* ≤ 0.01) (Figure 3). By sampling day, *Methanomethylophilus*, *UBA71*, and an unidentified genus within the *Methanomethylophilaceae* family increased in the rumen fluid while on E+ pastures and decreased after switching to non-toxic pasture, whereas *Methanobrevibacter_A* showed the opposite trend (Figure 4). The largest treatment-by-day differences (*p* ≤ 0.05) occurred on day 7. Over the study period, in rumen solids, *Methanobrevibacter_A* decreased while on E+ pastures and increased after the diet switch, whereas *UBA71* and an unidentified *Methanomethylphilaceae* genus showed the opposite pattern, with significant differences (*p* ≤ 0.05) mainly observed on days 7 and 14 (Figure 4).

Within E+ steers, no differences (*p* ≥ 0.2) were observed for any bacterial taxa (at genus level with RA ≥ 0.3%) between pre- and post-pasture placement in the rumen fluid, solid, or feces. However, in the rumen fluid, *Prevotella* showed an increase in RA from 15.8% pre to 28.3% post-pasture placement (*p* = 0.06), but not in the rumen solid (pre: 35.6%, post: 32.5%). Regarding the rumen fluid archaeal community, the RA of *Methanobrevibacter_A* decreased by approximately 23.5% following E+ pasture placement (pre: 84.3%, post: 64.5%, *p* = 0.07), whereas *UBA71* increased markedly, becoming almost 10 times higher over the same period (pre: 1.2%, post: 13.0%, *p* = 0.07). *Methanomethylophilus* also showed a markedly increased abundance following E+ pasture placement (pre: 0.0027%, post: 0.1975%), but this change did not reach statistical significance (*p* = 0.3). In the rumen solids, only *UBA71* increased following pasture placement, rising from 10.5% pre-treatment to 23.0% post-treatment, becoming approximately 2.2 times higher, although this change was only a strong trend (*p* = 0.06). In steers newly exposed to toxic pastures (second 14-day period), no significant changes in bacterial (*p* ≥ 0.2) or archaeal (*p* ≥ 0.4) RA were detected in any analyzed matrix after pre- and post-pasture comparisons.

### 3.6. Correlation Results

The only strong associations were observed between certain microbial genera in the rumen and feces and the concentration of lysergic acid in rumen fluid; however, these associations were only present during period 1 and absent in period 2. No significant associations were detected between rumen or fecal microbes (genus level) and EV concentrations or trace amine levels (tyramine, methyltyramine) in biological matrices. For more details, refer to Table 2.

## 4. Discussion

Exposure of steers to toxic fescue (E+) for 14 days did not alter alpha diversity indices significantly, indicating that the richness and evenness of the ruminal and fecal bacterial communities remained stable. This suggests a high resilience of the core microbiota to short-term exposure to E+ tall fescue grazing conditions. Consistent with this observation, no significant changes were observed in the relative abundance (RA) of the dominant taxa (≥0.3%), reinforcing the stability of major bacterial groups in the rumen and feces despite dietary challenges. However, not all microbial populations responded uniformly. Several low-abundance taxa (<0.3%) showed significant changes in E+ steers. Although these taxa represent a minor fraction of the microbial community, several belong to lineages with previously described metabolic capabilities that could be relevant to EA ingestion. This interpretation is further supported by the beta diversity analysis. While PERMANOVA detected significant differences in beta diversity, the associated R^2^ values were low (R^2^ = 0.04–0.07), indicating that treatment explained only a small proportion of the overall variation in community composition. Together with the extensive overlap observed in the ordination plots, these results suggest that the observed differences reflect relatively subtle shifts in community structure, likely involving changes in subdominant taxa rather than extensive restructuring of the dominant microbial community. Collectively, these results highlight the stability of the ruminal and fecal microbiome during short-term E+ fescue exposure, with minor taxa potentially contributing to functional adaptations in response to E+ pressure. Regarding the archaeal population, *Methanobrevibacter*, the dominant methane producer, decreased in both rumen fluid and solid fractions in E+ steers, while methylotrophic methanogens increased. This compositional pattern suggests that grazing E+ tall fescue may alter substrate availability or hydrogen flow in the rumen, driving changes in microbial community composition.

The ruminal microbial taxa with an RA of <0.3% that differed in E+ steers were dominated by the phylum *Bacillota*, suggesting that rare or low-abundance members of this phylum may play specialized roles in the EA-exposed rumen. Within this phylum, the class *Clostridia* dominated in both rumen fluid and solid. A previous study showed that ruminal hyper-ammonia-producing and tryptophan-utilizing bacteria, such as *Clostridium* spp., degrade ergovaline (EV) [10]. Although specific *Clostridium* species reported were not detected here, the presence of diverse Clostridia lineages supports their role in alkaloid metabolism. For example, taxa such as *CAG-269* (formerly annotated as *Clostridium* sp. in NCBI) in rumen fluid and solid and *Ruminiclostridium* (sp. *siraeum*) in rumen fluid increased in relative abundance (RA) in E+ steers. While less efficient than other species, *Prevotella* also demonstrated some capacity to degrade EV [10]. In E+ steers, *Prevotella* RA increased in rumen fluid, but not in the solid fraction post-pasture placement. Moreover, members formerly classified as *Prevotellaceae*, such as *UBA6382* and *Ga6A1*, were also increased in rumen fluid. The increase in *Prevotella* in the liquid, but not in the solid fraction, may be related to the fact that soluble EAs are more readily and rapidly available for metabolism by rumen microbes [7], which could preferentially influence the fluid-associated microbiota, and suggests that these taxa may play a role in EA metabolism. Overall, these findings are consistent with exposure to E+ tall fescue exerting a selective pressure that enriches rumen microbial lineages reported to possess detoxification capabilities, although such capabilities were not directly assayed in this study.

The functional capacity of ruminal microorganisms to detoxify plant-derived compounds has long been recognized [6]. Fermentation studies showed that toxic (E+) fescue seeds pre-incubated with rumen fluid are less toxic to rats than untreated seeds, indicating that the rumen microbiome may reduce toxicity [9]. Building on this, De Lorme et al. [34] and Hill et al. [35] reported that the rumen is the primary site for degradation and absorption of endophyte-derived alkaloids, and proposed that ergopeptine alkaloids are converted into simpler ergots, such as lysergic acid (LA), before absorption. Similarly, fermentation of endophyte-infected E+ fescue with viable rumen fluid caused a time-dependent increase in total EA, with EV a minor fraction, suggesting microbial transformation into other alkaloids, likely LA [8]. Ergot alkaloids share an ergoline ring structure, consisting of a LA core linked through an amide bond to a tripeptide moiety in the ergopeptines [36]. To reverse this process and liberate LA from the tripeptide, the peptide bonds must be hydrolyzed. The recovery of LA from rumen fluid [34] supports the idea that ruminal microbes possess this enzymatic capacity. These reactions rely on proteolytic activity; our study identified an enrichment of several low-abundance (<0.3%), but strongly proteolytic microbial taxa in E+ steers, including members of *Butyrivibrio_A* (e.g., *B. fibrisolvens*), *Eubacterium_G*, as well as members of the *Bacteroidaceae* family (*Ga6A1*, *UBA6382*; formerly classified within *Prevotellaceae*) [37,38,39]. Additionally, taxa with minor, though still evident, proteolytic activity were also enriched, e.g., members of the *Pseudomonadota* phylum [40], including the *Rhodocyclaceae* family (*Gammaproteobacteria*) and *Alphaproteobacteria*. Interestingly, in both rumen fluid and solid fractions, an unidentified genus belonging to *Alphaproteobacteria* was strongly positively correlated with rumen LA concentrations and warrants further investigation. In contrast, *Rhodocyclaceae*, despite increasing in E+ steers, was negatively correlated with LA during the second period. This does not necessarily contradict a proteolytic role: RA does not directly reflect enzymatic activity, and a negative association could arise if the LA released through proteolysis is rapidly further metabolized or absorbed, so that higher bacterial abundance coincides with lower measured concentrations. Given that this association was period-specific, it should be interpreted with caution. The benefit of ergopeptine catabolism by the rumen microbes may lie in their ability to use fragments of the cyclic tripeptide moiety (amino acids) for metabolism.

A key question in the ruminal metabolism of ergopeptines is the fate of amino acids released from the cleaved tripeptide moiety. Free amino acids in the rumen follow two routes: (1) incorporation into microbial protein [41] or (2) catabolism via decarboxylation or deamination to support microbial energy production [42]. Microbial protein synthesis requires sufficient carbon-derived energy [43]; however, in animals grazing E+ fescue, reduced feed intake [44] likely limits fermentable energy availability. Although direct feed intake measurement was beyond the scope of this study, we observed a reduction in fibrolytic taxa in the rumen fluid of E+ steers. Specifically, *Ruminococcus flavefaciens* (family *Ruminococcaceae*), a key microbe involved in fiber breakdown and energy release in the rumen [45], was significantly reduced. A similar decline in the genus *Ruminococcus* (*unidentified* sp.) was observed in rumen solids. This reduction in fibrolytic taxa, however, cannot be attributed to E+ tall fescue exposure alone. Because forage nutritive value (e.g., fiber content and composition) and individual feed intake were not measured in this study, the decline in *Ruminococcus* may reflect several non-mutually exclusive factors. On one hand, it could result from EA effects on rumen function, including decreased motility [46] and ruminal vasoconstriction [47], which may alter volatile fatty acid absorption [47] and, in turn, ruminal pH. On the other hand, steers grazing E+ fescue are well known to reduce their intake [44]; reduced intake by itself can alter the rumen environment (e.g., rumen fill, fiber substrate availability, and pH) [48], independently shaping the fibrolytic community. These intake- and forage-mediated effects are difficult to disentangle from a direct EA effect in the present design (see study limitations). Regardless of the underlying driver(s), this reduction in fibrolytic capacity may further contribute to the imbalance between nitrogen and energy availability.

Under energy-limited conditions, or when the rate of peptide breakdown exceeds that of amino acid assimilation, amino acid–derived nitrogen is redirected away from microbial protein synthesis and toward alternative pathways, including catabolism via decarboxylation or deamination [43,49]. This process could lead to the formation of trace amines (TAs) and biogenic amines (BAs) [50,51]. Here, within the low-abundance (<0.3%) but significantly affected microbes, several taxa previously described as possessing decarboxylase activity and the genetic potential to produce TAs and BAs were identified in E+ steers, including *Streptococcus* (*Streptococcaceae*) [52], *Butyrivibrio* [52,53], *Eubacterium* (*Lachnospiraceae*) [54], and *Selenomonas* (*Selenomonadaceae*) [55]. Interestingly, using an untargeted metabolomics approach on samples from the same study, TAs (e.g., tyramine, methyl-tyramine) and BA-like compounds (e.g., dopamine, serotonin) were detected in various biological matrices of E+ steers, including in rumen fluid [18]. These findings are consistent with previous grazing studies reporting TAs in rumen contents and urine of E+ steers [16,56], suggesting that EA exposure may divert amino acid utilization toward the synthesis of bioactive metabolites rather than microbial protein. It is important to mention that none of the TAs detected in rumen fluid were correlated either with the previously identified decarboxylase-producing bacterial taxa or with the other microbial genera found in rumen fluid and solid that were affected by E+. However, microbial 16S-rRNA abundance does not necessarily reflect enzymatic activity, which should be investigated further.

Another catabolic fate of some free amino acids under energy-limited conditions is the production of methylated compounds. These compounds in the rumen originate mainly from the fermentation of plant-derived substrates such as pectin and other methylated polysaccharides [57]. However, free amino acids such as methionine and cysteine can also be transformed by rumen microbes into methylated compounds [58]. In this study, the RA of microbes previously described as key contributors to methyl-compound formation in the rumen [57], such as *Butyrivibrio* and *Eubacterium*, increased in E+ steers. Consistent with these microbial shifts, an upregulation of methionine and cysteine metabolic pathways in the rumen fluid of E+ steers was observed in a parallel study [18]. Another potential methyl compound producer is *Prevotella*, which harbors enzymes (e.g., pectin methylesterases) that release methanol during plant fiber degradation [57]. In this study, the genus *Prevotella* and some of its members (e.g., *Ga6A1* and *UBA6382*) were enriched in the rumen of the E+ steers post-pasture placement. Collectively, these compositional findings, together with the metabolomic data from the companion study [18], are consistent with a scenario in which amino acid nitrogen is redirected towards the synthesis of TAs, BAs, and methylated compounds rather than microbial protein during E+ grazing.

Through fermentation, rumen microbes also produce methane, a gas that represents an energy loss for the animal (~2 to 12% of gross energy intake) and contributes to environmental pollution [14]. The primary methane producers are methanogenic archaea, which synthesize it from H_2_ and CO_2_. Besides this classical pathway, methane can also be generated from methylated compounds [59]. In our study, methylotrophic methanogens, including *UBA71*, an unclassified genus within the *Methanomethylophilaceae* family, *Methanomethylophilus*, and *Methanosphaera* showed increased abundance in the rumen fluid and solid of E+ steers during both periods. This finding aligns with a previous grazing trial that reported enrichment of methylotrophic methanogens, even with low EA levels in E+ pastures [16]. *Methanobrevibacter_A* is the dominant ruminal archaeal taxon, which consumes H_2_ and CO_2_ to produce methane. Although it remained the predominant archaeon, its RA decreased in rumen fluid and solid across both E+ grazing periods. This decline may reflect a shift in substrate availability following grazing of E+ tall fescue, favoring methylotrophic methanogens, and/or alterations in the rumen environment associated with E+ tall fescue exposure that suppress *Methanobrevibacter_A* or enrich microbes that compete with it for hydrogen. In line with this, other hydrogen competitors, in addition to methylotrophic methanogens, were also enriched. Specifically, members of the phylum *Bacillota*, order *Selenomonadales*, such as *Selenomonas ruminantium*, *Schwartzia succinivorans*, and *Centipeda*, were all enriched in both rumen fluid and solid of E+ steers. These bacteria have been described as a H_2_ sink that can utilize reducing equivalents such as H_2_ to produce propionate [60,61] and help maintain rumen redox balance. Consistent with this and across both study periods, the propionate concentration, as reported in a companion study, was significantly higher in the rumen fluid of E+ steers compared to the other groups [18]. Additionally, another H_2_ sink, valeric acid, was consistently elevated in the rumen fluid of E+ steers [18]. This should be considered in future research, as inhibition of methanogenesis has been previously associated with increased rumen valerate concentration [62,63]. Additionally, sulfate-reducing bacteria, such as an unclassified genus from the family *Desulfovibrionaceae* and a member of the genus *Desulfobulbus* from the *Desulfobulbaceae* family, also increased in the ruminal fluid of E+ steers. These microbes use H_2_ as an electron donor to reduce sulfate to hydrogen sulfide (H_2_S) [61]. It is important to note that their RA was low, suggesting that their overall contribution as a hydrogen sink may be limited, but their enrichment still reflects a reshaping of the ruminal ecosystem in response to E+ tall fescue exposure. It is worth highlighting that methane emissions were not measured in this study. The archaeal and microbial shifts described here are therefore interpreted as changes in substrate use and hydrogen flow within the rumen that may influence methanogenesis, and they cannot be taken as direct evidence of increased or decreased methane output. Overall, consuming E+ fescue may modify substrate availability and/or the ruminal environment, leading to adjustments or reshaping of rumen microbial communities that help sustain rumen function and fermentation processes, albeit at a potential energetic cost that may impair productivity.

Similar to the rumen microbiome, the core fecal microbiome was not altered in E+ steers. This suggests that 14 days of exposure may not be sufficient to induce detectable core microbiome changes. The fecal microbiome was dominated by *Bacillota* and *Bacteroidota*, consistent with previous findings [11,13]. In agreement with these reports, the most abundant families were *Oscillospiraceae* (formerly *Ruminococcaceae*) and *Bacteroidaceae*. Notably, no significant treatment effects were detected at the phylum or family level. This finding contrasts somewhat with an earlier study reporting modest phylum-level shifts (e.g., increases in *Planctomycetes*, *Chloroflexi*, and *Proteobacteria*) in the feces of E+ steers [11]. These differences most likely reflect biological factors, primarily the shorter exposure period in our study (14 vs. 28 days), which may have limited the extent of microbiome adaptation and/or disruption, and the contrasting season (fall vs. summer), which strongly influences forage growth, quality, and the resulting rumen and fecal microbial communities. Methodological differences, such as the use of OTUs [11] versus ASVs in the present study, may have contributed but to a lesser extent. At finer taxonomic resolution, only the genus *Paraprevotella* was significantly more abundant in E+ steers than in the other groups, aligning with Mote et al. [11]. As observed in the rumen, minor shifts in fecal low-abundance taxa (<0.3%) were observed, increasing during E+ grazing and returning to baseline afterward. For example, members of *Lachnospiraceae* (*Eubacterium*) and *Rikenellaceae* (*Mucinivorans*) exhibited this pattern. *Mucinivorans* is known as a mucin-degrading commensal that metabolizes mucus for its own growth [64], potentially affecting intestinal permeability. In contrast, members of the *Lachnospiraceae* family are often associated with butyrate production and intestinal homeostasis [65]; their transient increase is consistent with a possible compensatory response supporting gut function under dietary stress, although gut function was not measured. Overall, our findings show that grazing E+ fescue for 14 days was associated with subtle differences in the fecal microbiome, primarily in specific low-abundance bacterial groups, without differences in the overall fecal microbiome structure.

Throughout the second 14-day study period, no changes were observed in the RA of the top 15 bacterial taxa when comparing steers previously exposed to E+ to those never exposed. This was consistently evident across multiple taxonomic levels, including phylum, family, and genus. The absence of significant compositional changes in these most abundant members of the ruminal and fecal microbiota suggests that, under fall grazing conditions, prior EA exposure did not exert a persistent residual effect on the core rumen microbiome. This pattern aligns with the EA dynamics previously reported in these same animals [17], in which ruminal EV and LA concentrations declined within approximately 48 h of moving the steers off E+ pasture. The rapid clearance of these alkaloids, together with the concurrent recovery of the microbial community, indicates that once the alkaloid exposure was withdrawn, the dominant taxa returned to a composition indistinguishable from that of never-exposed animals. Taken together, the structure and stability of the dominant microbial community appear to be resilient to the challenge posed by short-term E+ exposure, at least during this specific seasonal window. It should be noted, however, that this conclusion applies specifically to the dominant (RA ≥ 0.3%) taxa; the persistence of low-abundance taxa (<0.3%) was not systematically assessed in this comparison, and residual effects on these rare members of the community cannot be excluded.

Several limitations should be considered when interpreting these findings. First, we did not measure forage nutritive value (e.g., NDF, ADF, crude protein, water-soluble carbohydrates), botanical composition, herbage mass, or individual feed intake. Because these factors are well-established drivers of rumen microbial structure [28,66], and because steers grazing E+ fescue typically reduce intake, we cannot fully disentangle effects attributable to EA from those mediated by forage quality, intake, rumen fill, or ruminal pH. The shifts reported here should thus be regarded as associated with E+ grazing rather than exclusively EA-driven, and the decline in fibrolytic taxa may partly reflect reduced intake and altered fiber availability. Second, with six steers per treatment, statistical power was limited, and the paired within-animal comparisons (*n* = 6) had reduced sensitivity; results reported as trends (0.05 < *p* < 0.10), along with the low-abundance taxa from FDR-corrected testing, should be treated as hypothesis-generating signals requiring confirmation in larger cohorts. Third, the assessment of residual effects was restricted to the high-abundance taxa (RA ≥ 0.3%); the persistence of low-abundance taxa was not systematically evaluated; additionally, potential changes in methane production were inferred from archaeal community composition rather than on direct measurement of methane emissions. Finally, microbial communities were characterized using 16S rRNA gene amplicon sequencing. While well established and cost-effective for resolving taxonomic composition and RA, this approach identifies which taxa are present rather than what they are actively doing. The metabolic roles discussed here (e.g., proteolysis, amino acid decarboxylation, methyl-compound formation, hydrogen utilization, detoxification) are therefore inferred from the documented capabilities of the identified taxa rather than demonstrated directly and should likewise be regarded as hypothesis-generating. Future work incorporating forage and intake measurements, larger groups, direct quantification of fermentation end-products and methane, and activity-based or meta-omics approaches (metagenomics, metatranscriptomics, or metabolomics) would help establish the causal and functional basis of the microbiota responses described here.

## 5. Conclusions

Short-term exposure (14 days) to toxic tall fescue (E+) did not alter the richness or evenness of the ruminal and fecal microbiota, demonstrating the resilience of the core microbial community. However, low-abundance taxa (<0.3%) responded selectively to E+ grazing conditions, a pattern consistent with functional adaptations within the microbiome based on the known metabolic capabilities of these affected taxa. In the rumen content, taxa reported to possess proteolytic, decarboxylase, and methyl-compound-producing capabilities increased in relative abundance, whereas taxa associated with fibrolytic activity decreased. This is consistent with the selective enrichment of bacterial taxa under E+ tall fescue grazing conditions, whose described metabolic functions may be advantageous in this environment. Changes in archaeal populations, including decreased hydrogenotrophic *Methanobrevibacter_A* and increased methylotrophic methanogens, along with enrichment of hydrogen-utilizing bacteria (e.g., *Selenomonas*), reflect not only adjustments in electron flow and rumen redox balance but also alterations in substrate availability under E+ grazing conditions. Collectively, these findings indicate that while 14 days of grazing E+ tall fescue does not disrupt the overall microbial structure, it may alter substrate availability and/or the rumen environment, reshaping low-abundance microbial lineages in a manner that, based on their previously described functions, may support rumen detoxification and help maintain fermentation homeostasis, potentially at an energetic cost. These functional interpretations are inferential, reflecting the taxonomic (16S rRNA) nature of the data, and warrant confirmation using activity-based or meta-omic approaches.

## Figures and Tables

**Table 1:** Low-abundance (<0.3%) microbial taxa differing significantly (*p* ≤ 0.05) between E+ and the rest in rumen and feces during one or both 14-day periods.

	Phylum	Class	Order	Family	Genus	Species	RA
Rumen fluid	*Bacillota A*	*Clostridia*	*Oscillospirales*	*CAG-272*	*UBA1248*	*–*	↓
	*Bacillota A* *	*Clostridia*	*Lachnospirales*	*Lachnospiraceae*	*Eubacterium G*	*–*	↑
	*Bacillota A* *	*Clostridia*	*Christensenellales*	*CAG-138*	*PeH17*	*PeH17*	↓
	*Bacillota A* *	*Clostridia*	*Lachnospirales*	*Lachnospiraceae*	*Butyrivibrio A*	*Fibrisolvens C*	↑
	*Bacillota A* *	*Clostridia*	*Lachnospirales*	*Lachnospiraceae*	*Butyrivibrio A*	*sp000621605*	↑
	*Bacillota A* *	*Clostridia*	*Oscillospirales*	*Ruminococcaceae*	*Ruminococcus C*	*Flavefaciens F*	↓
	*Bacillota A*	*Clostridia*	*TANB77*	*CAG-508*	*CAG-269*	*sp000431335*	↑
	*Bacillota I* *	*Bacilli_A*	*Lactobacillales*	*Streptococcaceae*	*Streptococcus*	*infantarius*	↑
	*Bacillota I*	*Bacilli_A*	*RF39*	*UBA660*	*UBA6985*	*sp900314465*	↑
	*Bacteroidota* *	*Bacteroidia*	*Bacteroidales*	*Bacteroidaceae*	*Ga6A1*	*–*	↑
	*Bacteroidota* *	*Bacteroidia*	*Bacteroidales*	*Bacteroidaceae*	*UBA6382*	*sp002439755*	↑
	*Pseudomonadota* *	*Gammaproteobacteria*	*Burkholderiales*	*Rhodocyclaceae*	*–*	*–*	↑
Rumen solid	*Bacillota A*	*Clostridia*	*–*	*–*	*–*	*–*	↑
	*Bacillota A*	*Clostridia*	*Lachnospirales*	*Lachnospiraceae*	*XBB2008*	*sp900102235*	↑
	*Bacillota A* *	*Clostridia*	*Lachnospirales*	*Lachnospiraceae*	*Butyrivibrio A*	*Fibrisolvens C*	↑
	*Bacillota A* *	*Clostridia*	*Lachnospirales*	*Lachnospiraceae*	*Butyrivibrio A*	*sp000621605*	↑
	*Bacillota A*	*Clostridia*	*Lachnospirales*	*Lachnospiraceae*	*G11*	*sp900103495*	↓
	*Bacillota A* *	*Clostridia*	*Oscillospirales*	*Ruminococcaceae*	*Ruminococcus C*	*–*	↓
	*Bacillota A*	*Clostridia*	*TANB77*	*CAG-508*	*UMGS1994*	*–*	↑
	*Bacillota A* *	*Clostridia*	*TANB77*	*CAG-508*	*CAG-269*	*sp000431335*	↑
	*Bacillota A* *	*Clostridia*	*Lachnospirales*	*Lachnospiraceae*	*Eubacterium G*	*–*	↑
	*Bacillota I*	*Bacilli_A*	*RF39*	*UBA660*	*RUG12783*	*–*	↓
	*Bacillota C* *	*Negativicute*	*Selenomonadales*	*Selenomonadaceae*	*Selenomonas B*	*Ruminantium A*	↑
Feces	*Pseudomonadota*	*Gammaproteobacteria*	*Burkholderiales*	*Rhodocyclaceae*	*–*	*–*	↑
	*Bacteroidota* *	*Bacteroidia*	*Bacteroidales*	*Bacteroidaceae*	*Paraprevotella*	*–*	↑
	*Bacillota I*	*Bacilli_A*	*RF39*	*UBA660*	*CAG-914*	*–*	↓
	*Bacillota A* *	*Clostridia*	*Lachnospirales*	*Lachnospiraceae*	*Eubacterium G*	*–*	↑
	*Bacillota A*	*Clostridia*	*TANB77*	*CAG-508*	*CAG-273*	*sp003507395*	↓
	*Bacillota A*	*Clostridia*	*Oscillospirales*	*UBA929*	*WRAI01*	*sp009780275*	↓

RA refers to relative abundance; arrows indicate direction of change (↑ = increased, ↓ = decreased). (*) indicates microbes that changed in the same direction during both the first and second 14-day periods and reached statistical significance (*p* ≤ 0.05) in at least one period.

**Table 2:** Repeated-measures correlation (rmcorr) analysis between microbial relative abundance (%) in the rumen and feces at the genus level and rumen ergot alkaloid concentrations of steers grazing toxic tall fescue during the first (Period 1) and second (Period 2) 14-day periods of the study.

	Class	Family	Genus	Species	Ergot Alkaloid	RA	Period 1 *r*	Period 1 *p*-Value	Period 2 *r*	Period 2 *p*-Value
Rumen fluid	*Alphaproteobacteria*	*UBA3830*	*UBA3830*	*sp902790255*	Lysergic acid	↓	0.9	<0.001	–	–
	*Alphaproteo* *bacteria*	*–*	*–*	*–*	Lysergic acid	↑	0.9	<0.001	–	–
	*Gammaproteobacteria*	*Rhodocyclaceae*	*–*	*–*	Lysergic acid	↑	–	–	−0.8	0.05
Rumen solid	*Clostridia*	*Lachnospiraceae*	*Kineothrix*	*Kineothrix alysoides*	Lysergic acid	↑	0.9	<0.001	–	–
	*Alphaproteo* *bacteria*	*–*	*–*	*–*	Lysergic acid	↑	0.9	<0.001	–	–
	*Alphaproteo* *bacteria*	*CAG-239*	*UBA1254*	*–*	Lysergic acid	↑	0.8	0.04	–	–
Feces	*Bacilli_A*	*Erysipelotrichaceae*	*Traorella*	*Traorella* *massiliensis*	Lysergic acid	↑	0.9	<0.001	–	–
	*Clostridia*	*Ruminococcaceae*	*Harryflintia*	*Harryflintia acetispora*	Lysergic acid	↑	0.9	0.01	–	–
	*Bacilli_A*	*Metamycoplasmataceae*	*–*	*–*	Lysergic acid	↑	0.8	0.02	–	–

Repeated-measures correlation (rmcorr) coefficients (*r*) ranging from −1 to 1 were calculated. Corresponding *p*-values indicate the statistical significance of each correlation (*p* ≤ 0.05). RA refers to relative abundance; arrows indicate direction of change (↑ = increased, ↓ = decreased).

## Data Availability

The dataset analyzed during the current study is available from the corresponding author on reasonable request. Accession number(s): All DNA sequences will be made publicly available in the NCBI Sequence Read Archive and will be accessible under BioProject accession number PRJNA1449026.

## Supplementary Materials

The following supporting information can be downloaded at: https://www.mdpi.com/article/10.3390/ani16162497/s1.
