# Supplementary material for: Microbiome Responses of Beef Steers to Rotational Grazing of Toxic Endophyte-Infected Tall Fescue Under Fall Conditions
Source: Animals (Basel). 2026 Aug 11;16(16):2497. doi: 10.3390/ani16162497 (PMC13508870; doi:10.3390/ani16162497)
Supplement: Supplementary file 1 [file animals-16-02497-s001.zip › animals-4457270-supplementary.pdf]

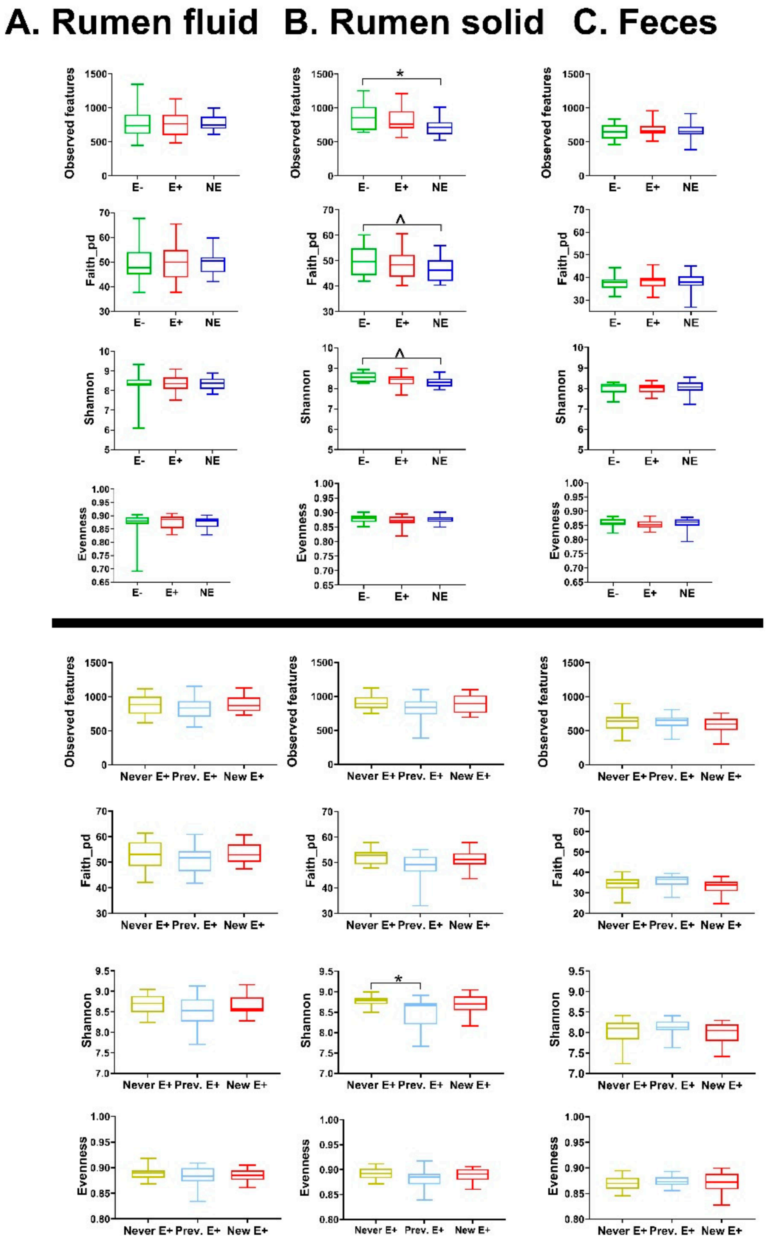

Figure S1: Alpha diversity metrics (number of observed features, faith's phylogenetic diversity index, shannon diversity index, and species evenness) in the A) rumen fluid, B) rumen solid and C) feces of Angus steers grazing toxic endophyte-infected (E+; n=6), non-toxic novel endophyte-infected (NE; n=6), and endophyte-free tall fescue E- (E-; n=6) during the first 14 days (upper panel), and after switching treatments (bottom panel). Data are presented as mean ± SEM; n = 6 animals per group. (*) indicates a significant difference between specific groups ($P \leq 0.05$), while (^) indicates trends ($P \geq 0.05$, $P < 0.1$).

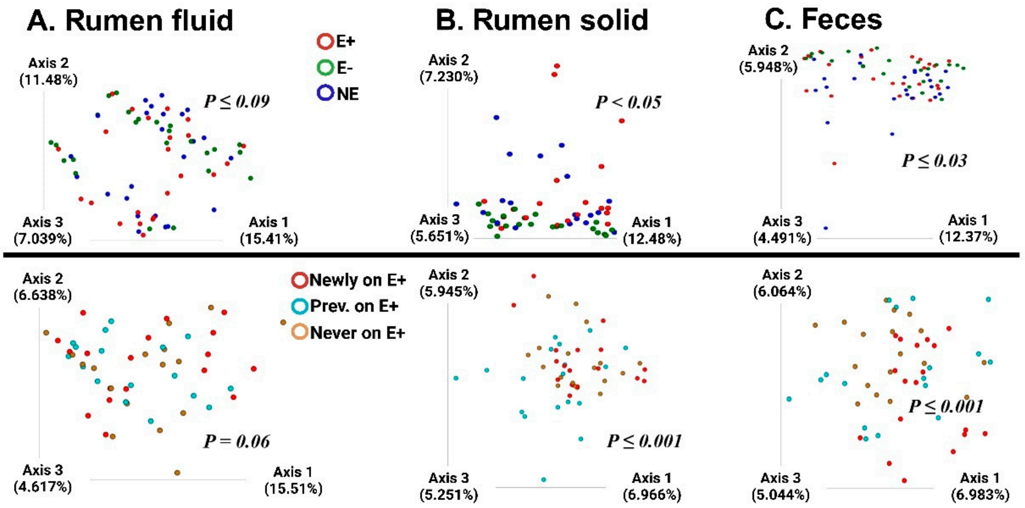

Figure S2: Principal coordinate analysis plot of beta diversity (Bray–Curtis dissimilarities) of bacterial population in the ruminal fluid A), solid B), and fecal C) samples of steers grazing toxic endophyte-infected (E+; n=6), non-toxic novel endophyte-infected (NE; n=6), and endophyte-free tall fescue (E-; n=6) during the first 14 days (upper panel), and after switching treatments (bottom panel). Significance determined at α=0.05.

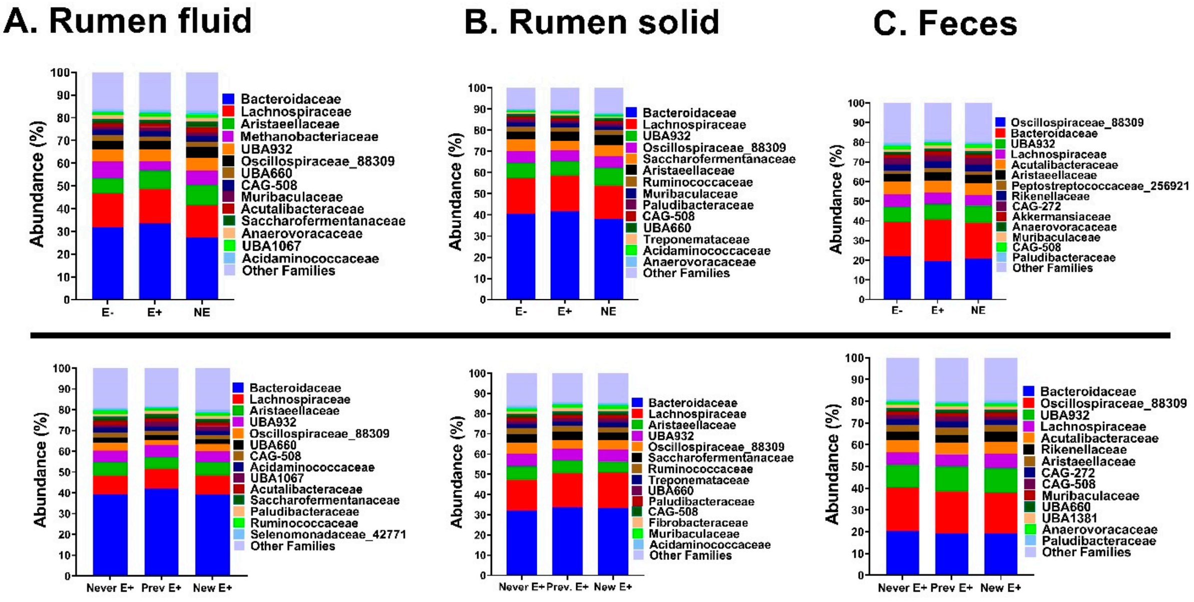

Figure S3. Relative abundance (%) of the top 15 families in the A) rumen fluid, B) rumen solid, and C) feces of Angus steers grazing on tall fescue infected with toxic endophyte (E+; n=6), novel non-toxic endophyte novel (NE; n=6), and endophyte-free (E-; n=6) during the first 14 days (upper panel), and after switching treatments (bottom panel).
